# Brain Morphology in Children with Epilepsy and ADHD

**DOI:** 10.1371/journal.pone.0095269

**Published:** 2014-04-23

**Authors:** Ricardo Saute, Kevin Dabbs, Jana E. Jones, Daren C. Jackson, Michael Seidenberg, Bruce P. Hermann

**Affiliations:** 1 Faculty of Medicine, Pontificia Universidade Catolica do Rio Grande do Sul (PUCRS), Porto Alegre, Brazil; 2 Department of Neurology, University of Wisconsin School of Medicine and Public Health, Madison, Wisconsin, United States of America; 3 Department of Psychology, Rosalind Franklin University of Science and Medicine, North Chicago, Illinois, United States of America; Tokyo Metropolitan Institute of Medical Science, Japan

## Abstract

**Background:**

Attention deficit hyperactivity disorder (ADHD) is a common comorbidity of childhood epilepsy, but the neuroanatomical correlates of ADHD in epilepsy have yet to be comprehensively characterized.

**Methods:**

Children with new and recent-onset epilepsy with (n = 18) and without (n = 36) ADHD, and healthy controls (n = 46) underwent high resolution MRI. Measures of cortical morphology (thickness, area, volume, curvature) and subcortical and cerebellar volumes were compared between the groups using the program FreeSurfer 5.1.

**Results:**

Compared to the control group, children with epilepsy and ADHD exhibited diffuse bilateral thinning in the frontal, parietal and temporal lobes, with volume reductions in the brainstem and subcortical structures (bilateral caudate, left thalamus, right hippocampus). There were very few group differences across measures of cortical volume, area or curvature.

**Conclusions:**

Children with epilepsy and comorbid ADHD exhibited a pattern of bilateral and widespread decreased cortical thickness as well as decreased volume of subcortical structures and brainstem. These anatomic abnormalities were evident early in the course of epilepsy suggesting the presence of antecedent neurodevelopmental changes, the course of which remains to be determined.

## Introduction

Attention deficit hyperactivity disorder (ADHD) is a common neuropsychiatric disorder in children and adolescents. The prevalence rate varies across individual studies, but is similar among pooled results regardless of the diagnostic procedure used and the countries or regions of the populations examined, ranging from 5.9 to 7.1% [1]. ADHD has substantial implications for quality of life in childhood as it is strongly associated with factors such as school performance, emotional adjustment, and peer relationships [2]. If persistent into adulthood, particularly if untreated, ADHD can be associated with lower socioeconomic status, work difficulties with frequent job changes, and other life problems [2].

Children with epilepsy have an increased prevalence of mental health disorders that include ADHD, mood and anxiety disorders, autistic spectrum disorder and conduct problems. These issues have been demonstrated repeatedly in epidemiological and clinical studies which have reported rates 4–5 times higher than the general population and 2.5 times higher than in individuals with other non-neurological chronic conditions [3]–[6]. ADHD in particular has been identified as a common comorbidity by several groups with rates varying from 21 to 31.5%—approximately 5 times higher than the general population [5]–[10].

Despite the elevated rate of ADHD in youth with epilepsy and its potential relevance for children's well-being [10], only a few neuroimaging investigations of this comorbidity have been published. It is not well established yet whether the mechanisms and neurobiological substrates of this disorder in children with epilepsy are similar to those in children without epilepsy. To our knowledge, the only examination of brain structure and ADHD in children with epilepsy was a prior analysis by our group [7] which examined total cortical lobar volumes as well as volumes of the cerebellum and brainstem. However, these analyses did not include examination of more contemporary metrics of cortical anatomy including thickness, area and curvature, nor a broader survey of subcortical structures, all of which are now possible with newer image processing systems which may yield new insights regarding the alterations in brain morphology associated with ADHD in children with epilepsy.

In the present study, we conducted a broad and comprehensive characterization of the neuroanatomical correlates of ADHD in childhood epilepsy. We included children with epilepsy with and without ADHD, as well as healthy controls, for the purpose of determining features specifically associated with ADHD that are not associated with epilepsy more generally. Additionally, in order to avoid confounding factors such as chronic seizure activity or long term anti-epileptic drug (AED) effects, as well as the cumulative social and psychological consequences of the disorder, we restricted our examination to children with new- and recent-onset epilepsy.

## Methods

### Participants

A total of 100 research participants comprised three groups of children: new and recent-onset epilepsy with ADHD (Epilepsy ADHD+, n = 18) and without ADHD (Epilepsy ADHD-, n = 36), and typically-developing first-degree cousin controls (n = 46). All participants were aged 8–18 years and attended regular schools. Children with epilepsy were recruited from the pediatric neurology clinics at three Midwestern medical centers (University of Wisconsin-Madison, Marshfield Clinic, Dean Clinic) and met the following inclusion criteria: (i) newly diagnosed epilepsy within the past 12 months; (ii) no other developmental disabilities (e.g. autism); (iii) no other neurological disorder, and (iv) normal clinical MRI. Children with epilepsy underwent routine MRI as part of their diagnostic workup and all scans were interpreted as unremarkable by the clinical neuroradiologists. This point was later confirmed by the research pediatric neurologist when reviewing the cases for study participation. Inclusion in the Epilepsy ADHD+ group required a diagnosis of ADHD according to DSM-IV [12] criteria (see *Assessment of ADHD* below). Exclusion criteria for controls were: (i) any initial precipitating event (e.g. simple or complex febrile seizures); (ii) any seizure or seizure-like episode; (iii) diagnosed neurological disease; (iv) loss of consciousness greater than 5 min; or (v) other family history of a first-degree relative with epilepsy or febrile convulsions; (vi) ADHD. Further demographic and clinical features of the three groups are provided in Table 1. Table 1 shows that there were 25 children/adolescents with localization-related epilepsies (LRE) and 29 with idiopathic generalized epilepsies (IGE). Of the total epilepsy group, 7 were on no medications, 46 were on monotherapy and 1 was on polytherapy. There were no differences between the ADHD+/− groups and demographic features (age, gender) or epilepsy characteristics (age of onset, duration of epilepsy, number of medications, epilepsy syndrome). IQ was lower in the Epilepsy ADHD+ group compared to the Epilepsy ADHD- and Controls groups, but nevertheless fell in the average range.

**Table 1:** Demographic and clinical characteristics of participants (means and standard deviations).

Variable	Control (n = 46)	Epilepsy ADHD- (n = 36)	Epilepsy ADHD+(n = 18)^a^
Age (y)	13.08 (3.26)	13.47 (3.41)	12.11 (3.15)
Sex (% female)	27 (58%)	20 (56%)	7 (39%)
FSIQ	110.20 (11.21)	109.81 (11.81)	93.44 (9.23)*
Seizure onset age (y)	—	12.56 (3.37)	11.02 (3.26)
Epilepsy duration (m)	—	8.58 (3.72)	8.33 (3.05)
AED's (0/1/2+)	—	6/30/0	1/16/1
Syndrome (IGE/LRE)	—	21 (58%)/15 (42%)	8 (44%)/10 (56%)
ADHD medication (1+)	—	—	7

a ADHD subtypes: Inattentive (n = 9); Hyperactive-Impulsive (n = 3); Combined (n = 3); NOS (n = 3).

FSIQ: Full-scale intelligent quotient.

AED: Anti-epileptic drug.

IGE: Idiopathic generalized epilepsy.

LRE: Localization-related epilepsy.

*p<0.05 compared to Control and Epilepsy ADHD- groups.

First-degree cousins were used as controls rather than siblings or other potential controls groups because: (i) first-degree cousins are more genetically distant from the participants with epilepsy and thus less pre-disposed than siblings to shared genetic factors that may contribute to anomalies in brain structure and cognition; (ii) a greater number of first-degree cousins were available than siblings in the target age range and (iii) the family link was anticipated to facilitate participant recruitment and especially retention over time (which is our intent) compared to more general control populations (e.g. unrelated school mates).

This project represents an extension of our previous report [7] but includes several distinguishing features. The sample is partially but not completely overlapping as some T1 only scans were unacceptable for current analysis compared to the prior investigation that used T1, T2 and proton density (PD) weighted images—thus more recent participants with acceptable T1 scans were included. The neuroimaging analyses are completely distinct and comprehensive in scope as will be described below. Finally, the epilepsy control group (ADHD-) is quite different. In order to have an “uncomplicated” epilepsy group, we selected children not only without ADHD, but without reported academic problems as well (e.g., special learning services, tutors, grade retention). Since academic problems are often comorbid with ADHD, and they exist in a significant proportion of children with epilepsy without ADHD, it was felt to be important to eliminate these children from the epilepsy control group (ADHD-).

### Assessment of ADHD

To determine ADHD status the Kiddie–Schedule for Affective Disorders and Schizophrenia–Present and Lifetime Version (K-SADS-PL) was used [13]. This is a semi-structured diagnostic interview designed to assess current and previous episodes of psychopathology in children and adolescents using DSM-IV criteria [12]. The KSADS-PL has been utilized in several studies of children with epilepsy to identify rates of psychiatric comorbidity including ADHD [14], [15]. The K-SADS-PL was administered by interviewing both the parent and the child separately. In the current study, two interviewers were specially trained to administer the clinical interview, and interviews were randomly selected for review to ensure diagnostic consistency. Fifteen percent of participants were randomly selected for independent review to ensure reliability of diagnoses and reduce rater drift. Interviewers were not blinded to seizure history as this often arose spontaneously during the interview. There was no association between ADHD+/− and epilepsy syndrome (IGE vs. LRE) (p = 0.34), age of onset (p = 0.12), duration of epilepsy (p = 0.80), or AED number p = 0.13).

### MRI acquisition

Images were obtained on a 1.5T GE Signa MRI scanner (GE Healthcare, Waukesha, WI, U.S.A.). Sequences acquired for each participant were T1-weighted, three-dimensional (3D) spoiled gradients recall (SPGR) using the following parameters: TE = 5 ms, TR = 24 ms, flip angle  = 40 degrees, NEX = 1, slice thickness  = 1.5 mm, slices  = 124, plane  =  coronal, field of view (FOV)  = 200 mm, matrix = 256×256. All MR images were inspected before image processing. Image quality was rated on a five-point scale (0 = poor 4 = excellent) and we required a minimum quality rating of 3 or better for the scan to be included in this analysis. Further details regarding MRI processing are provided in the supporting information file Methods S1.

### Human Subjects

This study was reviewed and approved by the Institutional Review Boards of all participating institutions (University of Wisconsin-Madison, Marshfield Clinic, Dean Clinic). On the day of study participation families and children gave informed consent and assent, respectively. All procedures were consistent with the Declaration of Helsinki [11]. The investigators obtained informed written consent from the next of kin, caretakers, or guardians on behalf of the minors/children participants involved in the study. Those study participants age 18 completed written informed consent.

### Analyses

Analyses focused on differences between the Epilepsy ADHD+, Epilepsy ADHD- and control groups in regard to cortical thickness, area, volume and curvature, as well as volumes of subcortical and brain stem structures. For cortical measurements, a surface-based group analysis using Freesurfer's statistical tool Qdec was performed. The participants' surface data were smoothed using 10, 15 or 20 mm FWHM kernels to optimize significant regions. A general linear model was then applied at each vertex to test for group differences in cortical thickness, area, volume and curvature. Age and gender were modeled as covariates for all measures while intracranial volume (ICV) was added as a covariate for measures of cortical area, volume and curvature. To correct for multiple comparisons, a Monte-Carlo simulation was implemented with an initial cluster-forming threshold set to p<0.05. Clusters were then tested against an empirical null distribution of maximum cluster size built using synthesized Z-distributed data across 10,000 permutations, producing cluster-wise p-values (CWP) fully corrected for multiple comparisons. A False Discovery Rate (FDR) of p<0.05 was also independently applied to correct for multiple comparisons. Finally, we examined subcortical structure volumes previously reported to be abnormal in epilepsy and/or ADHD using multivariate analysis of covariance (MANCOVA), with age, gender and ICV as covariates.

## Results

### Cortical thickness (vertex analysis)

**Figure 1:**
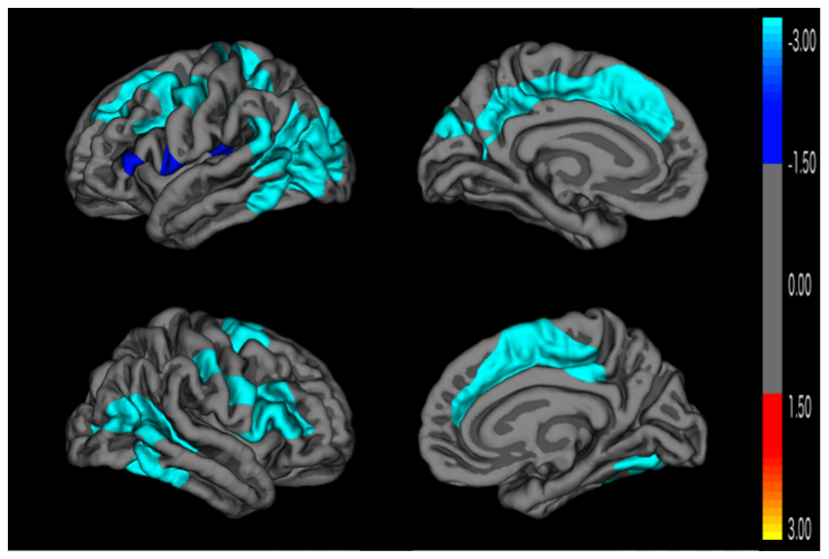
Cortical thickness comparison between epilepsy ADHD+ and control groups. Monte Carlo cluster-wise simulation: Blue colors indicate significant clusters where the Epilepsy ADHD+ group is thinner than the control group.

Figure 1 and Table 2 depict the results of the Monte Carlo simulation comparing Epilepsy ADHD+ to healthy controls. All surviving significant (CWP<0.05) clusters represent areas of decreased cortical thickness in the Epilepsy ADHD+ group versus healthy controls. Affected regions in the left hemisphere include: 1) caudal and rostral middle frontal areas, superior aspect of the frontal lobe, pre and postcentral gyri, superior and inferior aspects of the parietal lobe, supramarginal gyrus, cuneus, lateral aspect of the occipital lobe; and posterior areas of inferior, middle and superior aspects of the temporal lobe and banks of the superior temporal sulcus; 2) paracentral lobule, precuneus, posterior and caudal anterior aspects of the cingulate cortex, and superior medial aspect of the frontal lobe; and 3) pars triangularis, pars opercularis, lateral extremity of the pre and postcentral gyri, insula, and transverse temporal gyrus. Affected areas in the right hemisphere include: 1) inferior aspects of the parietal lobe, lateral aspects of the occipital lobe, fusiform gyrus, and posterior areas of inferior, middle and superior aspects of the temporal lobe and banks of the superior temporal sulcus; 2) pars opercularis, pars triangularis, rostral and caudal middle frontal areas, pre and postcentral gyri, and supramarginal gyrus; and 3) superior aspect of the frontal lobe, caudal middle frontal area, paracentral gyrus, and rostral anterior, caudal anterior and posterior aspects of the cingulate cortex.

**Table 2:** Regions of significant differences in vertex analyses of cortical thickness (CT) and cortical area (CA).

Comparison Groups	Region of Interest	Cluster size (mm^2^)	P corrected	Tal. Coord. (x y z)
CT: Epi ADHD+ vs. Control	L superior parietal	18015.46	0.0001	−23.4 −76.1 18.4
	L paracentral	4909.15	0.0001	−18.0 −31.7 37.8
	L insula	2336.06	0.0188	−31.0 −24.0 13.5
	R inferior parietal	5444.41	0.0001	37.9 −56.8 17.5
	R pars opercularis	5207.25	0.0001	41.4 7.9 18.6
	R superior frontal	5277.92	0.0001	7.6 −2.5 53.3
CT: Epi ADHD+ vs. Epi ADHD-	L inferior parietal	8318.56	0.0001	−38.0 −48.8 33.5
	L caudal middle frontal	3843.39	0.0001	−40.2 14.9 44.7
	L posterior cingulate	1972.37	0.0436	−13.1 −33.5 38.3
	R pars opercularis	6338.68	0.0001	44.0 21.1 16.4
CA: Epi ADHD- vs. Control	L middle temporal	1320.29	0.0457	−59.0 −36.3 −11.1

**Figure 2:**
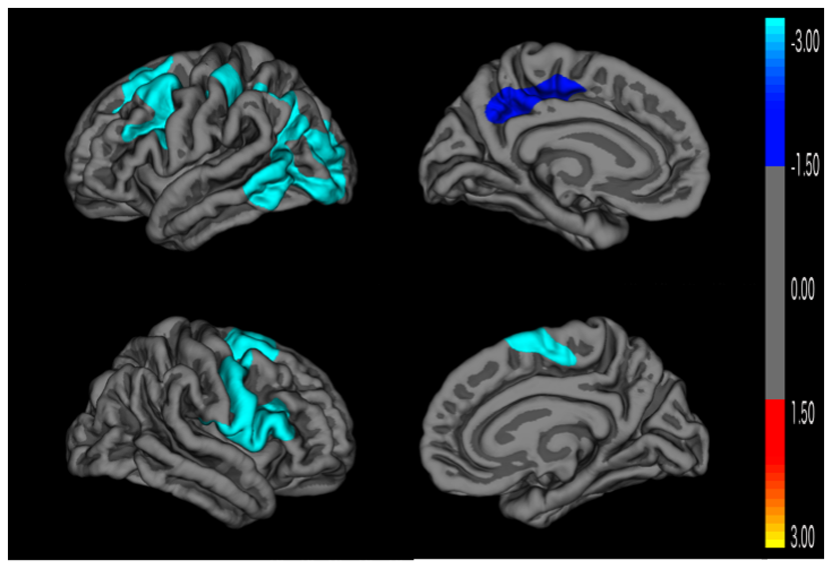
Cortical thickness comparison between Epilepsy ADHD+ and Epilepsy ADHD- groups. Monte Carlo cluster-wise simulation: Blue colors indicate significant clusters where the Epilepsy ADHD+ group is thinner than the Epilepsy ADHD- group.

Figure 2 depicts the results of the Monte Carlo simulation comparing Epilepsy ADHD+ to Epilepsy ADHD- children. The Epilepsy ADHD+ group exhibits significant cortical thinning in several left hemisphere regions including: 1) inferior and superior aspects of the parietal lobe, post and precentral gyrus, supramarginal gyrus, lateral aspect of the occipital lobe, and inferior and middle aspects of the temporal lobe; 2) precentral gyrus, pars opercularis, and caudal middle, rostral middle and superior aspects of the frontal lobe; and 3) posterior cingulate cortex, precuneus, paracentral lobule, and superior medial aspect of the frontal lobe. The Epilepsy ADHD+ group also exhibits a cluster of decreased thickness in the right cerebral cortex, including: 5) pars opercularis, pars triangularis, rostral and caudal middle frontal areas, pre and postcentral gyri, supramarginal gyrus, superior aspect of the frontal lobe, and paracentral lobule. There were no cortical thickness differences between the Epilepsy ADHD- and controls after correction for multiple comparisons.

### Cortical surface area, volume and curvature (vertex analysis)

**Figure 3:**
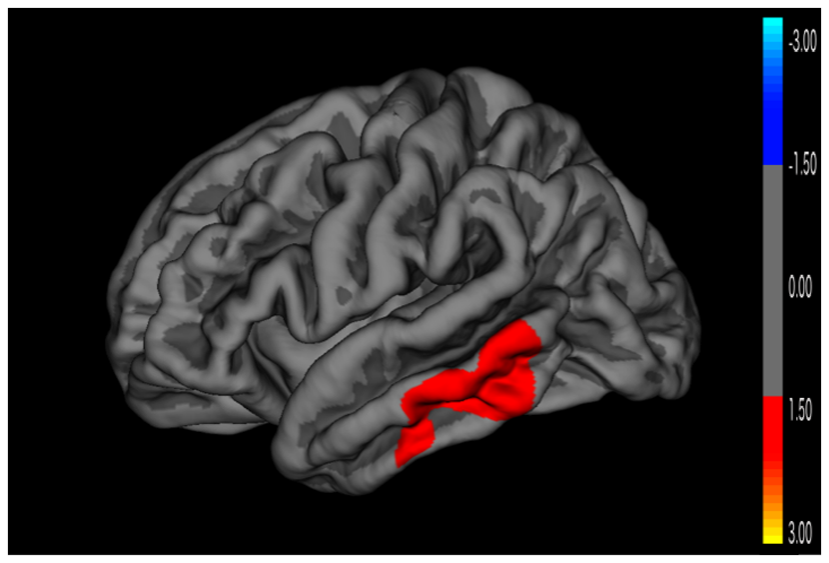
Cortical area comparison between Epilepsy ADHD- and control groups. Monte Carlo cluster-wise simulation: Red colors indicate significant clusters where the Epilepsy ADHD- group is larger in area than the control group.

Figure 3 reveals a significant cluster of greater surface area in Epilepsy ADHD- compared to healthy control groups in the left middle and inferior temporal regions. There were no differences in area in comparisons involving the Epilepsy ADHD+ group. Analyses of cortical volume did not yield any significant difference among groups after correction for multiple comparisons. Cortical volume is the product of two independent components, cortical thickness and surface area. In the case where one is increasing and the other is decreasing the volume change can be zero.

Cortical surface curvature assesses differences in small areas (peak of gyri and sulci), making FDR a more appropriate correction. The curvature index is zero for a locally flat surface and the magnitude increases as the surface becomes more spiked. Sulci and gyri are distinguished by positive and negative curvatures, respectively. Minor dissimilarities surviving FDR are found in two group comparisons (supporting information file Figure S1). Epilepsy ADHD+ participants demonstrate a small region of increased curvature in the lateral orbitofrontal cortex and an area of decreased curvature in the fusiform gyrus, compared to epilepsy ADHD- participants. Epilepsy ADHD- participants exhibit increased curvature compared to controls in three regions: 1) the inferior portion of the parietal lobe, 2) posterior cingulate cortex, and 3) precuneus; as well as a small area of decreased curvature at the isthmus of the cingulate cortex.

### Subcortical volumes

*A s*ummary of the volumetric analyses of subcortical structures and cerebellum is provided in Table 3. Three patterns of results can be appreciated. First, an arguably strong ADHD association was characterized by greater abnormality (reduced volume) in the Epilepsy ADHD+ participants compared to *both* the Epilepsy ADHD- and the control groups, who did not differ from one another. The right caudate showed this degree of specificity. Second, a more common pattern of association was characterized by greater abnormality in the ADHD+ compared to *either* the ADHD- *or* the control group, with the ADHD- and control groups again not differing from one another. Regions of interest demonstrating this pattern included total subcortical grey matter, brainstem, left caudate, left thalamus, and right hippocampus. Third, there was a general relationship of epilepsy characterized by both epilepsy groups (ADHD+ and ADHD-) differing significantly from the controls, with the epilepsy groups not differing from each other. Regions of interest showing this pattern included volumes of the right thalamus, and total cerebellum (due to left cerebellum).

**Table 3:** Subcortical volume comparisons between groups.

Association	Region of Interest	Control	Epilepsy ADHD-	Epilepy ADHD+
ADHD	R caudate	4052	4063	3770* #
	Total subcortical GM	203171	199105	195759*
	Brainstem	20818	20587	19767*
	L caudate	4024	4015	3800*
	L thalamus	7744	7622	7487*
	R hippocampus	4404	4434	4191#
Epilepsy	R thalamus	8082	7846*	7631*
	Cerebellum	149152	144560*	143296*
	L cerebellum	74389	71890*	71411*

*p<0.05 compared to control group.

# *p*<0.05 compared to Epilepsy ADHD- group.

## Discussion

In this study a comprehensive set of analyses were performed to characterize alterations in cortical morphology, subcortical structures, and cerebellum that may be associated with ADHD in children with epilepsy. By incorporating children with epilepsy with and without ADHD, we were able to identify anatomical abnormalities associated with epilepsy more generally versus abnormalities associated more specifically with ADHD. Three core findings emerged from this investigation: 1) ADHD in children with epilepsy is associated with a distributed pattern of anatomic abnormality involving the cortex and subcortical structures, the cortical morphological abnormalities characterized overwhelmingly by reduced cortical thickness in bilateral areas of the frontal, parietal, and temporal lobes, with minor or no group differences in metrics of cortical surface area, volume or curvature; 2) Children with epilepsy and ADHD exhibited significantly smaller subcortical volumes of the caudate, thalamus, hippocampus, and brainstem; 3) A more general association with epilepsy, unrelated to the presence or absence of ADHD, was seen in reduced volumes of the cerebellum and thalamus. These findings will be reviewed below.

First, the main finding is a diffusely distributed pattern of neuroanatomical abnormality in children with epilepsy and ADHD compared to children with epilepsy but without ADHD and typically-developing controls. These results involve broad and widespread cortical and subcortical areas suggesting associations between altered brain networks in ADHD that participate in executive function, attention, and sensorimotor systems [16]. The Epilepsy ADHD+ group exhibited significant cortical thinning in areas related to the dorsal attention network including the left intraparietal sulcus, left superior areas of the parietal lobe, superior frontal and precentral gyrus; as well as in ventral attention network areas such as the supramarginal gyrus and the temporoparietal junction in the left and the inferior frontal gyrus in the right hemisphere [17], supporting more general developmental ADHD findings. Cortical thinning is also the notable characteristic of ADHD in the general population [18]–[28].

We found minimal differences in cortical area or volume when examining the Epilepsy ADHD+ group. Nevertheless, two studies have reported decreased cortical area in children with ADHD compared to controls [29], [30]. Curvature analysis revealed very modest differences between children with epilepsy with versus without ADHD, these findings restricted to two small regions and not emerging in the comparison between Epilepsy ADHD+ and controls, indicating that this may be an incidental finding of uncertain clinical significance. Shaw et al. also found no differences in their gyrification index (a measure comparable to curvature) between ADHD and controls [30].

Second, at the subcortical level, children with epilepsy and ADHD differed significantly from the Epilepsy ADHD- and control groups in the volume of the right basal ganglia, as well as from either the Epilepsy ADHD- or the control group, with the latter two groups comparable to each other across volumes of the left caudate, left thalamus, right hippocampus, and brainstem, with additional reduced volume of total subcortical grey matter. Several of these results are consistent with meta-analyses and reviews of ADHD in the general population [31]–[35]. These studies were based primarily on structural MRI differences using voxel-based morphometry between children with ADHD and healthy controls, with the most constant findings involving smaller right caudate [31], [34], [35], right putamen and right globus pallidus [31], [33], [35] in children with ADHD. Left caudate [31] volume has also been shown to be decreased, although with less consistency across studies. Additionally, abnormalities in shape analyses – which we did not examine in the present investigation – have been reported in the basal ganglia, hippocampus and amygdala in children with ADHD [32]. These meta-analyses have not identified changes in thalamus, probably because of the relatively small number of investigations targeting this structure and the lack of segregation of specific thalamic nuclei [36]. However, two recent publications provide new insights regarding the potential role of thalamus morphometry in ADHD. Ivanov and colleagues demonstrated significantly smaller regional volumes bilaterally in the pulvinar nuclei in children with ADHD compared to healthy controls despite their finding of no significant difference in total thalamus volume. Moreover, those individuals treated with stimulant medication exhibited pulvinar measurements more similar to controls than those under no treatment [37]. Further, Xia and colleagues reported reduced volume in the right and left thalamus in youth with ADHD in comparison to healthy controls, although this finding was not significant in the left hemisphere when controlling for age and gender. Examining subdivisions within the thalamus, significant volume differences were found in the ventral anterior, medial dorsal, and pulvinar nuclei in the left but not right thalamus [38]. These results, together with our finding of diminished left thalamic volume in ADHD, are consistent with anatomic evidence implicating the thalamus in cortical-striatal-thalamic-cortical loops that are significant substrates for higher cognitive functions, including attention [39].

Brainstem volume, likewise, is understudied in the ADHD literature, but our result is physiologically reasonable, given that the brainstem hosts the reticular activating system and is the production site for neurotransmitters with a key role in ADHD [40], [41]; and there is reported probable involvement of this structure in attention manifestations of various neuropsychiatric disorders [42]. Cerebellar atrophy has long been considered a consequence of epilepsy chronicity/severity or the effects of specific medications, namely phenytoin [43], but here we find cerebellar atrophy in children with new onset (and not chronic) epilepsy and no children had been prescribed or taken phenytoin. This result was driven largely by volumetric reduction in the left cerebellum. The volume of the left thalamus follows the same pattern, indicating an association with epilepsy as well. In contrast to our results, one of the most robust regions of decreased volume in a ROI based meta-analysis of general ADHD was the cerebellum, in particular the posterior inferior vermis [34], but here no such effect was found. Cerebellar volume reduction is a recurrent finding in the epilepsy literature, even though it seems to be more prominent in chronic cases [44]–[48]. Accordingly, thalamic volume is shown to be decreased bilaterally in investigations of both focal and generalized epilepsy syndromes [26], [49]–[51].

In the epilepsy ADHD literature several clinical seizure features, including seizure frequency [52], clinical control of seizures [52], number of antiepileptic drugs [7], and duration of epilepsy [7], [9], are reported as unassociated with the frequency of ADHD diagnosis or reported symptoms. ADHD studies in epilepsy samples usually detect more inattentive subtype than in developmental ADHD [7], [9], [53], [54]. Finally, ADHD symptoms in children with epilepsy have been reported to improve with stimulant medication in in the same way they do in the general population [54]–[57].

In summary, this study indicates that ADHD, a common comorbidity in pediatric epilepsy, is associated with thinning of cortical regions and diminution of subcortical structures related to attention, executive function and sensorimotor networks.

There are differences in the neuroimaging results of this investigation compared to our previous report [7] which might be due to number of factors. First and foremost, the epilepsy control group (ADHD-) is substantially different and more rigorously defined. To derive a clearly “uncomplicated” epilepsy group, we selected children not only without ADHD but also without reported academic problems as well, that is, children who had been provided with a variety of academic services or academic adjustments for achievement problems. These included special school-based learning services, tutors, grade retention, formal individual education plans, and other provisions. Since academic problems are often comorbid with ADHD, and they exist in a significant proportion of children with epilepsy without ADHD, we felt it appropriate to exclude these children from the epilepsy control group (i.e., ADHD-). Second, our previous study utilized an entirely different image processing system (Brains2) which combined T1, T2 and PD images for tissue segmentation supplemented by VBM to measure gray matter volume, while the current study examines gray matter surface based features (thickness, area, volume and curvature). Although these two measurements of gray matter volume in general are correlated, differences can exist as each imaging technique indexes different biological signals [58]. Volume based VBM observes the proportion of gray matter voxels, based on signal intensity threshold, compared to voxels representing other tissue type such as white matter and cerebrospinal fluid (CSF) [59]. As such, gray matter volume measured in VBM can be sensitive to changes in gray matter and CSF volumes as well as differences in cortical surface curvature, which cannot be distinguished from each other. Gray matter thickness measures the distance between white-gray matter boundary to cortical surface, which is less affected by adjacent CSF volume and cortical curvature and primarily reflects packing density and arrangement of neuronal cells [60]. In summary, distinctions in cohort characteristics between the current and our previous study likely influenced the neuroimaging findings with additional contribution from differences in morphometric analysis techniques. Note that neuroimaging findings in the current study are in line with the general ADHD literature, which have consistently shown cortical thinning with a similar anatomical distribution.

### Limitations, Strengths, and Future Directions

Our study has both limitations and strengths. Regarding limitations, first noted is that the number of children with epilepsy and ADHD is modest. Second, we were not able to include an ADHD group without epilepsy as too few controls met criteria for ADHD, thereby precluding a true 2×2 analysis. Third, because of the modest sample size, it was not possible to perform analyses examining ADHD as a function of the known subtypes including inattentive, hyperactive, and combined—a very important task for the future. Fourth, the mechanisms that underlie the ADHD comorbidity in epilepsy remain an open question. This investigation does not inform that point. One avenue we are currently investigating is whether family history/family aggregation of ADHD is a pertinent consideration. Despite these limitations, the strengths of the paper include that fact that ADHD is an important complication of childhood epilepsy, the diagnosis of which was defined rigorously based on DSM-IV diagnosis from the K-SADS-PL rather than a parent report questionnaire which thereby improved diagnostic reliability. We included measures of cortical morphology that went beyond cortical thickness, measures that have been understudied in epilepsy in general and epilepsy and ADHD in particular. Because of that, we felt that there was utility in examining these measures comprehensively and indeed it appears that alterations in cortical thickness are a primary correlate of ADHD in epilepsy. Moving forward, we are tracking these children prospectively (2 years and 5-6 years later) and plan to focus on their longitudinal trajectories in the future.

## Conclusions

ADHD is a common co-occurring condition in childhood epilepsy. This comorbidity of epilepsy has anatomical correlates that are detectable early in the course of the epilepsy suggesting that the altered neuroanatomy is not due to epilepsy chronicity or treatment. The etiology of this anatomical signature and its prospective course remain to be characterized, as does the life course of children with epilepsy with ADHD compared to children with epilepsy without ADHD.

## Supporting Information

Figure S1 **Cortical curvature comparisons.** A) FDR corrected significant differences in curvature. Blue colors indicate regions in which the Epilepsy ADHD+ group has decreased curvature relative to the Epilepsy ADHD- group (fusiform region). Red colors indicate regions in which the Epilepsy ADHD+ group has increased curvature relative to the Epilepsy ADHD- group (lateral orbitofrontal prefrontal region). B) FDR corrected significant differences in curvature in the right hemisphere. Blue colors indicate regions in which the Epilepsy ADHD - group has decreased curvature compared to the control group. Red colors indicate regions in which the Epilepsy ADHD - group has increased curvature compared to the control group.
(TIF)

Methods S1 **MRI processing details.**
(DOCX)
