# Supplementary figures and images for: Brain Morphology in Children with Epilepsy and ADHD
Source: PLoS One. 2014 Apr 23;9(4):e95269. doi: 10.1371/journal.pone.0095269 (PMC3997349; doi:10.1371/journal.pone.0095269)

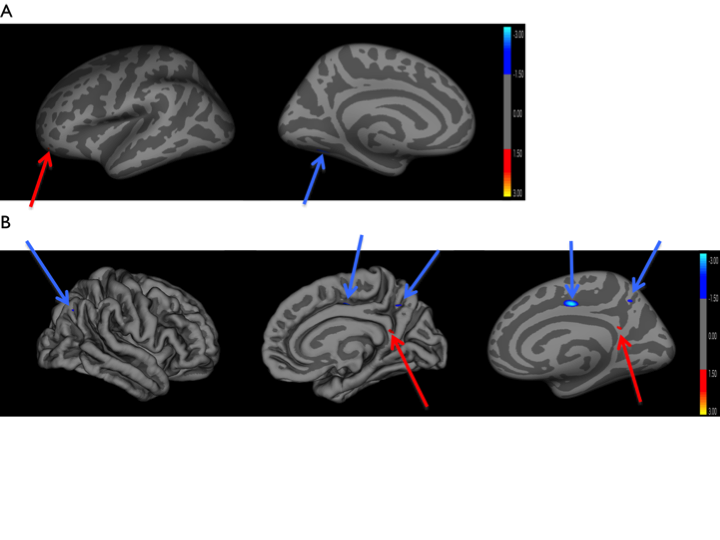

Supplement: Figure S1 — Cortical curvature comparisons. A) FDR corrected significant differences in curvature. Blue colors indicate regions in which the Epilepsy ADHD+ group has decreased curvature relative to the Epilepsy ADHD- group (fusiform region). Red colors indicate regions in which the Epilepsy ADHD+ group has increased curvature relative to the Epilepsy ADHD- group (lateral orbitofrontal prefrontal region). B) FDR corrected significant differences in curvature in the right hemisphere. Blue colors indicate regions in which the Epilepsy ADHD - group has decreased curvature compared to the control group. Red colors indicate regions in which the Epilepsy ADHD - group has increased curvature compared to the control group. (TIF) [file pone.0095269.s001.tif]
