# Supplementary material for: Brain Morphology in Children with Epilepsy and ADHD
Source: PLoS One. 2014 Apr 23;9(4):e95269. doi: 10.1371/journal.pone.0095269 (PMC3997349; doi:10.1371/journal.pone.0095269)
Supplement: Methods S1 — MRI processing details. (DOCX) [file pone.0095269.s002.docx]

**Supporting Information**

**Methods S1**

*MRI processing details*

Images were transferred to a Mac OSX (Apple Inc., Cupertino, CA, U.S.A.) computer for processing with the FreeSurfer image analysis suite, which is documented and freely available for download online (http://surfer.nmr.mgh.harvard.edu/). FreeSurfer is a set of software tools for the study of cortical and subcortical anatomy. The T1 volumetric MRI scans were used for cortical reconstruction and volumetric segmentation. The technical details of these procedures are described in prior publications [1-8]. Briefly, this processing includes removal of nonbrain tissue using a hybrid watershed/surface deformation procedure [6], automated Tailarach transformation, segmentation of the subcortical white matter and deep gray matter volumetric structures (including hippocampus, amygdala, caudate, putamen, ventricles) [9, 10], intensity normalization [11], tessellation of the gray matter-white matter boundary, automated topology correction [4, 12], and surface deformation following intensity gradients to optimally place the grey-white and gray-cerebrospinal fluid (CSF) borders at the location where the greatest shift in intensity defines the transition to the other tissue class [1-3]

After establishment of the white matter and pial surfaces, voxel-wise measurements of cortical thickness, area, volume and curvature are generated. Thickness is calculated as the closest distance from the gray-white boundary to the grey-CSF boundary at each vertex on the tessellated surface [3]. Gyral and sulcal features are then used to delineate surface curvature. Comparison of surface measures across participants is possible after surface inflation [13], and registration to a spherical atlas, which uses individual cortical folding patterns to match cortical geometry across participants [14]. The spherical atlas naturally forms a coordinate system in which point-to-point correspondence between participants can be achieved. Mean thickness differences between groups can then be displayed on the pial surface of the standard atlas. An automated parcellation of the cerebral cortex into units based on gyral and sulcal structure [10, 15] results in 34 neuroanatomical regions per hemisphere (4 medial temporal, 5 lateral temporal, 11 frontal, 5 parietal, 4 occipital, and 4 cingulate). Average thickness, area and volume measures are produced for each of the parcellation regions of interest.

The data presented here were processed using the FreeSurfer version 5.1 default processing stream (recon-all –all). Data were visually inspected, and manual interventions were performed when the automated steps reported errors or failed quality assurance review. These included manual alignment to the Talairach template in cases where the automatic registration was wrong and adjustments to the watershed threshold to restore areas of the brain that were erroneously removed during skull stripping. If the automatic subcortical segmentation did not label voxels correctly, editing of the gray matter and white matter segmentation image was completed when defects were evident on routine visual inspection/quality control. Several of the Freesurfer programs use Talairach coordinates as seed points. In general, if the tissue segmentation or surfaces did not pass internal QA inspection the registration to the atlas was examined. The image was then manually aligned to the atlas and the processing was restarted. Gray matter edits can be performed if the subcortical segmentation does not label voxels correctly.

**References**

[1] Dale AM, Sereno MI (1993) Improved localization of cortical activity by combining EEG and MEG with MRI cortical surface reconstruction: a linear approach. J Cogn Neurosci 5: 162-176.

[2] Dale AM, Fischl B, Sereno MI (1999) Cortical surface-based analysis. I. Segmentation and surface reconstruction. Neuroimage 9: 179-194.

[3] Fischl B, Dale AM (2000) Measuring the thickness of the human cerebral cortex from magnetic resonance images. Proceedings of the National Academy of Sciences of the United States of America 97: 11050-11055.

[4] Fischl B, Liu A, Dale AM (2001) Automated manifold surgery: constructing geometrically accurate and topologically correct models of the human cerebral cortex. IEEE Trans Med Imaging 20: 70-80.

[5] Fischl B, van der Kouwe A, Destrieux C, Halgren E, Segonne F, et al. (2004) Automatically parcellating the human cerebral cortex. Cerebral Cortex 14: 11-22.

[6] Segonne F, Dale AM, Busa E, Glessner M, Salat D, et al. (2004) A hybrid approach to the skull stripping problem in MRI. Neuroimage 22: 1060-1075.

[7] Han X, Jovicich J, Salat D, van der Kouwe A, Quinn B, et al. (2006) Reliability of MRI-derived measurements of human cerebral cortical thickness: the effects of field strength, scanner upgrade and manufacturer. Neuroimage 32: 180-194.

[8] Jovicich J, Czanner S, Greve D, Haley E, van der Kouwe A, et al. (2006) Reliability in multi-site structural MRI studies: effects of gradient non-linearity correction on phantom and human data. Neuroimage 30: 436-443.

[9] Fischl B, Salat DH, Busa E, Albert M, Dieterich M, et al. (2002) Whole brain segmentation: automated labeling of neuroanatomical structures in the human brain. Neuron 33: 341-355.

[10] Fischl B, Salat DH, van der Kouwe AJ, Makris N, Segonne F, et al. (2004) Sequence-independent segmentation of magnetic resonance images. Neuroimage 23 Suppl 1: S69-84.

[11] Sled JG, Zijdenbos AP, Evans AC (1998) A nonparametric method for automatic correction of intensity nonuniformity in MRI data. IEEE Trans Med Imaging 17: 87-97.

[12] Segonne F, Pacheco J, Fischl B (2007) Geometrically accurate topology-correction of cortical surfaces using nonseparating loops. IEEE Trans Med Imaging 26: 518-529.

[13] Fischl B, Sereno MI, Dale AM (1999) Cortical surface-based analysis. II: Inflation, flattening, and a surface-based coordinate system. Neuroimage 9: 195-207.

[14] Fischl B, Sereno MI, Tootell RB, Dale AM (1999) High-resolution intersubject averaging and a coordinate system for the cortical surface. Hum Brain Mapp 8: 272-284.

[15] Desikan RS, Segonne F, Fischl B, Quinn BT, Dickerson BC, et al. (2006) An automated labeling system for subdividing the human cerebral cortex on MRI scans into gyral based regions of interest. Neuroimage 31: 968-980.
